# ICCVAM: Birnbaum and Stokes Respond

**DOI:** 10.1289/ehp.1001969R

**Published:** 2010-05

**Authors:** Linda S. Birnbaum, William S. Stokes

**Affiliations:** National Institutes of Health, Department of Health and Human Services, Research Triangle Park, NC, USA, E-mail: birnbauml@niehs.nih.gov; NIEHS, National Institutes of Health, Department of Health and Human Services, Research Triangle Park, North Carolina, E-mail: stokes@niehs.nih.gov

Sandler’s comments about our editorial concerning the Interagency Coordinating Committee on the Validation of Alternative Methods (ICCVAM) (Birnbaum and Stokes 2010) suggest a lack of awareness of the role and significance of the contributions of ICCVAM. The 2008 *Washington Post* article she cites (Gaul 2008) contained many inaccurate statements (a letter correcting the errors was submitted to the *Washington Post,* but it was not published). We appreciate this opportunity to provide accurate factual information about ICCVAM.

ICCVAM is a congressionally mandated committee that does not have laboratories and does not develop test methods or conduct validation studies. Rather, ICCVAM depends on other organizations, including its 15 member agencies, to carry out such activities. The director of the National Institute of Environmental Health Sciences (NIEHS) established ICCVAM in 1997, with the cooperation of 14 other agencies, in order to provide a coordinated interagency process to facilitate the regulatory acceptance of scientifically valid alternative methods. As an interagency forum, ICCVAM also coordinates and promotes related issues, including national and international harmonization, guidance on validation studies, and awareness of accepted alternative methods.

ICCVAM was formally established by legislation in 2000 with signing of the ICCVAM Authorization Act of 2000. This law charges ICCVAM to “review and evaluate new or revised or alternative test methods, … including the coordination of technical reviews of proposed new or revised or alternative test methods ….” ICCVAM develops and submits recommendations based on its reviews to the Secretary of Health and Human Services for transmittal to federal agencies. Agencies must review the recommendations and respond to ICCVAM within 180 days. ICCVAM has implemented a transparent and scientifically rigorous evaluation process for test methods that has resulted in national and international regulatory acceptance of all recommended test methods. ICCVAM has contributed to the acceptance of 33 alternative test methods, including 17 based on formal comprehensive evaluations (ICCVAM 2010). Recommendations on an additional 4 methods are pending.

The National Toxicology Program (NTP) Interagency Center for the Evaluation of Alternative Toxicological Methods (NICEATM) administers ICCVAM and provides scientific and operational support for ICCVAM activities. Consistent with the NTP mission, NICEATM also conducts independent validation studies on new, revised, and alternative test methods, and coordinates international validation studies with its counterparts in Japan, Europe, and Canada (NIEHS 2009).

In 2008, NICEATM and ICCVAM launched a 5-year plan to further reduce, refine, and replace the use of animals in regulatory testing in conjunction with federal agencies and other stakeholders (ICCVAM 2008). The plan seeks to advance alternative test methods of high scientific quality that will continue to protect and advance the health of people, animals, and the environment. The plan emphasizes using new technology to develop predictive systems that will lessen or avoid the need for animals where scientifically feasible.

The NIEHS and NTP support research that may lead to the development of new test methods relevant to regulatory testing. These include the Tox21 collaboration between the NTP, the National Institutes of Health Chemical Genomics Center, and the U.S. Environmental Protection Agency (Schmidt 2009). The Tox21 initiative is the largest *in vitro* toxicology research program ever conducted worldwide and is expected to yield candidate methods and approaches with potential applicability to regulatory testing. Following standardization and validation in consultation with ICCVAM, methods with regulatory applicability will be reviewed by ICCVAM and recommendations forwarded to appropriate agencies.

ICCVAM has been enormously successful in gaining regulatory acceptance of alternative methods (ICCVAM 2010). Gaining regulatory acceptance requires high-quality studies that prove that the alternative test methods will provide the same or better level of protection of workers and consumers as the methods they might replace. The test method must also be shown to be reproducible in different laboratories.

The animal welfare benefits of ICCVAM’s work are evidenced by many examples. These include an alternative test for acute oral toxicity that has replaced the LD_50_ test (median lethal dose), which used as many as 200 animals per test, with the Up-and-Down Procedure (UDP), which uses only 7 animals on average per test (NIEHS 2001; Organisation for Economic Co-operation and Development 2008). The UDP and other alternative test methods have profoundly reduced animal use for acute oral toxicity testing, which is conducted to determine the poisoning potential of chemicals and products and is the most commonly conducted safety test worldwide.

Another landmark ICCVAM contribution is the reduction and refinement of animal use for eye-safety testing. ICCVAM evaluated and recommended the first two *in vitro* test methods that can now be used to determine whether substances can cause blindness and other severe eye damage, without the need for live animals (NIEHS 2008). Based on ICCVAM’s evaluation, these test methods were adopted as international test guidelines in 2009.

In summary, ICCVAM has demonstrated its effectiveness and value in achieving the regulatory acceptance of test methods that reduce, refine, and replace animal use. Most importantly, by making appropriate science-based decisions, ICCVAM has ensured that such methods will continue to protect the public’s health and safety. We expect ICCVAM to serve an increasingly important role in translating research advances into improved test methods that will benefit both people and animals.
